# On the Production of Cataract in Frogs by the Administration of Sugar

**Published:** 1860

**Authors:** S. Weir Mitchell


*On the Production of Cataract in Frogs by the Administration of Sugar.* By S. WEIR MITCHELL, M. D. (Read before the Biological Department of the Academy of Natural Sciences, October 3, 1859.

A few months ago I had occasion to perform a large number of experiments upon the osmosis of woorara through animal membranes. During one of these experiments, a solution of the poison was placed within the stomach of a rabbit, and, the two extremities of the organ being secured by ligature, it was suspended in syrup. At the close of two hours a portion of the syrup, about two drachms, was injected into the subcutaneous tissues of a pigeon, who did not suffer from it in any way. A frog of small size received at the same time, in the dorsal subcuticular sac, about one and a half drachms of the suspected syrup. Much to my surprise he became feeble, and died in about four or five hours. As it was not impossible that the syrup used might contain woorara, owing to this substance having exosmosed from the stomach, the death of the frog was attributed to the poison. To correct this result, the remainder of the syrup, about three ounces, was evaporated to dryness and treated with alcohol. The alcohol was then carefully evaporated to a small bulk, and injected under the skin of a pigeon. Upon close examination it did not appear to be poisoned, or to be in any way injured by the injected material. It now occurred to me that, as the amount of sugar employed in the case of the frog was very great compared to his bulk, it might, possibly, be destructive to life when used in very large doses.

*Experiment.*—To test this view, three frogs of middle size were treated with sugar in the form of syrup, two drachms being injected into the dorsal subcuticular sac of each animal. Within two hours the first frog became sluggish; the dorsal sac, which had gradually enlarged, swelling from an accumulation of fluid in its interior. This fluctuating mass was the syrup, augmented by the exosmosis of fluids from the vessels or extra-vascular tissues, or both. As the frog became more and more feeble, the larger part of this collection of fluid disappeared. The frog died at the close of ten hours. During the latter portion of this time my attention was arrested by the white appearance of the frog's eyes, which, on close examination, proved to be cataractous; the cornea remaining perfectly clear and transparent.

*Experiment.*—The second frog was treated with repeated doses of syrup given internally. The phenomena of exosmosis were far less marked in this case, but large quantities of mucus collected in both stomach and œsophagus, and were finally disposed of, in part, by vomiting. The mode of death did not differ from that last described. The cataractous appearance was not seen until just after the frog's death.

*Experiment.*—The third frog was placed in syrup, so that when seated in the usual posture the syrup covered its hind legs. Death took place in seventeen hours, but the cataract was not formed as usual, or, at least, was not externally visible. I did not examine its eyes post-mortem.

From the time I observed the development of this curious form of cataract it became a chief point of interest in the sugar poisoning, and was studied with attention. A few preliminary experiments convinced me that the best and most certain mode of causing the cataract was to inject the sugar in syrupy solution into the subcuticular sacs. The results thus obtained were extremely curious. Of eight frogs, nearly alike as to size, and all of one species (*R. pipiens*,) thus treated with injections of two and a half drachms, six had cataract. In four of the six this was apparent during life, in one it was doubtful until after death, and in one no cataract could be seen until after death. Of the cataractous cases one was thus affected short of six hours, the remainder became so affected between six and thirty-eight hours. Two of the frogs suffered considerably from the poisoning, if such it may be termed; but both survived, and had no externally visible cataractous opacity. In all of these experiments the frogs were placed under bell glasses, tilted to insure ventilation, and were kept moist in each case by a piece of wetted sponge. In a second series of experiments, conducted in precisely a similar manner, it was found that when the frog died very early, as sometimes occurred, no cataract became visible. When they survived rather longer, the cataract was a more frequent incident; but in a few cases no such formation took place, in despite of frequent doses of sugar to a very large amount.

It now became clear to me that I had discovered a method of producing in frogs an opacity of the crystalline lens, which might be of some value as illustrating the pathology of a subject which has always been one of extreme difficulty. So far as I am aware, no one has ever succeeded in causing cataract in the eyes of dead animals without wounding the organ, and all hope of being able to govern its synthesis in living animals has long since been given up. Many of the frogs upon whom I operated survived the constitutional effects, and remained more or less active with highly cataractous lenses. The change produced was not, therefore, of necessity associated with mortal symptoms, nor could it be regarded as a mere *post-mortem* phenomenon, since, even in the animals which did not finally survive, the lens became opaque some time before death.

With the view of ascertaining the cause of the opacity of the lens produced by the sugar poisoning, certain experiments were directed towards the determination of the effect of altering the external conditions while the frog was still suffering from the sugar. The first experiment was as follows:

*Experiment.*—A large frog received under his skin about two drachms of syrup. Two drachms were also forced into the stomach through a tube and the same amount was given in a similar manner at the close of an hour. As soon as the frog became sluggish he was placed in water and left there. He soon began to recover, and the water about him being changed thrice in the ensuing eight hours, he recovered perfectly. The dose of sugar would certainly have sufficed to destroy life had the supply of water been limited. A repetition of the last experiment satisfied me that, even with a very large dose of sugar, the animal was safe if allowed to remain in water kept fresh by frequent changes. Thus far everything pointed to osmotic changes as the probable agents in the production of the curious variety of cataract under consideration. The result of the next experiment, which in the sequence of thought naturally suggested itself as a test of this hypothesis, was such as to strengthen it considerably.

*Experiment.*—About two drachms of syrup were injected under the skin of a large frog. In twenty-four hours the lens was opaque, and, as the animal appeared lively, it was placed in water in order to test the permanence of the opacity. Ten hours in the water sufficed to remove most of the opacity from the lens, which began to clear in the centre first. Twenty-four hours after the frog had been placed in water the eyes were perfectly transparent, and the animal itself entirely well.

*Experiment.*—A distinct cast of double cataract was produced in a large frog by the usual means. When the cataract first began to be visible it was placed in water. During five hours the opacity increased. In the ensuing eight hours it diminished perceptibly, but, although the water was changed twice a day, some traces of the cataract were visible during several days. The frog recovered entirely from all the effects produced by the sugar.

*Experiment.*—A large frog was seated in syrup. In a few hours he was nearly dead. The mucous membrane of the mouth and tongue was intensely congested, and the parts under the eye particularly so. On placing the animal in water he slowly recovered. The eyes remained clear throughout, and were not visibly affected. The congestion above referred to is a constant accompaniment of the death by sugar, but varies in degree to a remarkable extent, and does not seem to be in any way connected with the alteration of the lens.

It was found in the course of the several experiments related, when syrup was thrown into the subcuticular dorsal sac of a frog, it at first acquired increased bulk, owing to the rapid osmosis of their fluids from the frog's tissues. During this period no change occurred in the lens. As the saccharine solution became more and more diluted, the current of interchange developed, and the sugar gradually soaked into the tissues of the frog, so as to be found in most of the subcuticular sacs as well as in the peritoneal cavity. As it was still possible that the original loss of water by the tissues during the first stage of sugar poisoning, might be the cause of the cataract which afterwards formed, and, as in this case, the effects produced would be in some respects similar to rapid desiccation, the following simple test was employed:—

*Experiment.*—Two frogs were placed in open jars and allowed to remain without water, the temperature being from 75° to 88° F. During the experiment one frog died, on the fifth day, the other on the sixth day. In neither of them was there any cataract. Both frogs were much shrunken from the loss of fluid. Mere desiccation was, therefore, insufficient to cause the opacity.

From time to time, during the conduct of these experiments, the lenses of the poisoned frogs were carefully examined by Dr. Hewson, Dr. Hunt, and myself. Dr. Hunt very kindly furnished me with notes of his observations, and, as they accord perfectly with my own results, I shall content myself with quoting his description of the general appearances presented by the cataractous lenses: "The capsule of the lens is clear, and the cells upon the lenticular surface are unaltered. The opacity begins upon the posterior face of the lens directly in the axis of vision. It is next seen on the anterior surface around a clear central spot, which corresponds to the line of cleavage between the different systems of lens fibres or tubes, the centre of the star of the lens. The opacity gradually extends all around the lens, but as yet I have never met with a case where it involved the central portions; which, on the contrary, always remain clear, notwithstanding this limitation. The outside color of the lens is often of a pearly whiteness, and the simulation of a true cataract is absolutely perfect."

When such a lens is viewed under a low power, in place of the faint indication of the track of the lens fibres which is usually seen, the line of cleavage is unduly distinct, and the fibres setting out from it are edged with dark, irregular lines, marking the interlocking with the neighboring fibres. A good deal of granular matter is also dispersed through the preparations. In more advanced cases the fibres or tubes are enlarged irregularly, and, their interior contents escaping, are seen abundantly in the form of yellowish pellucid globules about the tubes and throughout the field of view. My friend, Dr. Hunt, and I have also observed that the same changes may be produced by soaking the eyes of frogs in syrup. By properly regulating the strength of the syrup, cataract map be thus induced without any rupture of the eyeball. I have made no experiments with larger eyes, but it is probable that, in these, also, cataract could be thus induced, and the eyes then made use of to teach the operative manual. Some such resource has long been considered desirable by teachers of ophthalmic surgery.

It may be further remarked that opacity by sugar may be produced by simply soaking the exposed lens in sugar and water. However caused, the cataractous whiteness disappears when the lenses are placed in water, but they do not become entirely transparent where the opacity has existed for some time, or where it is very highly marked. This may be owing to the fact that in extreme cases the lens tubes are not merely altered in form, and in their relations to one another, but are also ruptured and partially emptied of their softer albuminous contents; lesions which no restoration of their aqueous supply could entirely relieve.

It appears, from the various experiments here related, that mere abstraction of water from the lens is insufficient to cause opacity; a conclusion which is strengthened by the knowledge that the exposed lens, when dried, does not become opaque. As it is found that the formation of the cataract attends the second stage of sugar poisoning, or that in which the sugar soaks into the tissues, it is probable that the direct contact of sugar with the lens is essential to the production of the phenomenon in question. That the changes which then result are osmotic seems sufficiently clear; but whether due chiefly to absorption of sugar in solution by the crystalline humor, or to exosmosis of the thinner portions of the lens fluids to the sugar, we have no means of determining. We may conclude, however—*First.* That sugar in large amounts destroys the life of the frog when given internally, injected under the skin, or thrown into the stomach. *Second.* That an abundant supply of water frequently enables the frog to eliminate the sugar and escape death. *Third.* That the formation of a peculiar variety of cataract is one of the most curious and striking symptoms attendant upon the sugar poisoning. *Fourth.* That the cataract is due to mechanical disturbances of the form and relative position and contents of the component tubes of the lens.

It is, perhaps, unnecessary to remark here that we have no knowledge of any such form of cataract in man. Notwithstanding this, it would be improper to omit to state that cataract has occasionally been found to co-exist with advanced diabetes mellitus. Very recently, indeed, Mr. France[1] has reported five cases of double cataract occurring in diabetic cases. In all of these, the cataract, which was always soft, formed with great rapidity when the constitutional malady was far advanced; and in all of them the lens increased in size antero-posteriorly, and the opacity attacked portions of several strata of the crystalline humor at once, leaving clear and transparent interspaces. Now that the diabetes has any other causative relation to the cataracts in question than through the general impairment of the nutritive functions common in this disease, I do not pretend to say; but as it is possible that the long-continued presence of even a small

[1] American Journal of the Medical Sciences, July, 1859, p. 266; from Ophthalmological Hospital Reports, Jan. 1859.

amount of sugar in the blood may cause in the crystalline lens osmotic changes productive of opacity, I have felt it proper to call attention anew to the relation between the two maladies in question.

